# Dr. Mynter’s Letter

**Published:** 1886-12

**Authors:** 


DR. MYNTER'S LETTER.—We regret to say that an interesting letter from our friend Dr. Mynter, from Copenhagen, has been crowded out of the last two numbers of the JOURNAL. The Doctor is in excellent health and making the most of his time in study and research.

